# Reconciling Oil Palm Expansion and Climate Change Mitigation in Kalimantan, Indonesia

**DOI:** 10.1371/journal.pone.0127963

**Published:** 2015-05-26

**Authors:** Kemen G. Austin, Prasad S. Kasibhatla, Dean L. Urban, Fred Stolle, Jeffrey Vincent

**Affiliations:** 1 Nicholas School of the Environment, Duke University, Durham, North Carolina, United States of America; 2 World Resources Institute, Washington, DC, United States of America; Montana State University, UNITED STATES

## Abstract

Our society faces the pressing challenge of increasing agricultural production while minimizing negative consequences on ecosystems and the global climate. Indonesia, which has pledged to reduce greenhouse gas (GHG) emissions from deforestation while doubling production of several major agricultural commodities, exemplifies this challenge. Here we focus on palm oil, the world’s most abundant vegetable oil and a commodity that has contributed significantly to Indonesia’s economy. Most oil palm expansion in the country has occurred at the expense of forests, resulting in significant GHG emissions. We examine the extent to which land management policies can resolve the apparently conflicting goals of oil palm expansion and GHG mitigation in Kalimantan, a major oil palm growing region of Indonesia. Using a logistic regression model to predict the locations of new oil palm between 2010 and 2020 we evaluate the impacts of six alternative policy scenarios on future emissions. We estimate net emissions of 128.4–211.4 MtCO_2_ yr^-1^ under *business as usual* expansion of oil palm plantations. The impact of diverting new plantations to low carbon stock land depends on the design of the policy. We estimate that emissions can be reduced by 9-10% by extending the current moratorium on new concessions in primary forests and peat lands, 35% by limiting expansion on all peat and forestlands, 46% by limiting expansion to areas with moderate carbon stocks, and 55–60% by limiting expansion to areas with low carbon stocks. Our results suggest that these policies would reduce oil palm profits only moderately but would vary greatly in terms of cost-effectiveness of emissions reductions. We conclude that a carefully designed and implemented oil palm expansion plan can contribute significantly towards Indonesia’s national emissions mitigation goal, while allowing oil palm area to double.

## Introduction

Meeting growing demand for food, fiber and fuel while minimizing environmental degradation is a critical societal challenge [1,2]. Global agricultural production will need to at least double in order to match demand from a rapidly growing population, putting acute and widespread pressure on natural resources [3,4]. Climate change is one of the most significant global impacts of agriculture. The Intergovernmental Panel on Climate Change reports that agriculture and land use change contribute one quarter of global greenhouse gas (GHG) emissions [5]. Mitigating these emissions while simultaneously achieving increases in food production will require a departure from *business as usual* (BAU), including improved land use management practices [6].

Indonesia, which has the highest rate of primary forest loss and the fourth highest GHG emissions in the world, exemplifies this global challenge [7,8]. In 2010, the nation embarked on a strategy to reduce GHG emissions by 26% unilaterally, and up to 41% with international support, below a projected baseline by 2020 [9]. The majority of pledged emissions reductions will be accomplished by reducing deforestation and forest and peat land degradation. These conditions are driven in large part by conversion for oil palm and timber plantations, which contribute more than three quarters of national GHG emissions [10,11].

Indonesia has also pledged to grow its agricultural sector, including doubling palm oil production by 2020 [12]. Palm oil is the most abundant vegetable oil globally, with production increasing more rapidly than any other oil crop in the world [13]. Indonesia produces more than 50% of global palm oil, and its 2011 palm oil exports generated 19 billion USD [14]. Since 2000 more than 70% of expansion in oil palm cultivation occurred at the expense of peat lands and primary, secondary, and agro forests, leading to significant greenhouse gas (GHG) emissions [15–17].

If past trends continue, oil palm expansion will jeopardize Indonesia’s GHG reduction commitment. In 2011, in an effort to improve governance and management of forest resources, the government announced a moratorium on all new concessions in primary natural forests and peat lands [18]. In addition, several provincial governments have designed low-emissions development strategies, which include mitigating negative impacts of agricultural expansion, including diverting expansion from high conservation value forest and peat lands to low carbon and low biodiversity land [19,20]. More recently, several palm oil companies have committed to eliminate deforestation and peat land conversion from their supply chains. As of February 2015, more than 90% of the world’s internationally traded palm oil was bound by these commitments [21].

The objective of this study is to quantify the potential CO_2_ emissions reductions achievable via the extension of the current moratorium to the year 2020 and the implementation of low-emissions land use strategies by the oil palm industry in Kalimantan (Indonesian Borneo). This region contributes one quarter of Indonesia’s current palm oil production, and the Indonesian Ministry of Forestry has identified it as a priority area for oil palm expansion [14,22]. Carlson et al. [16] estimate that oil palm expansion in Kalimantan contributed 3–12% of total Indonesian GHG emissions during 2000 – 2010.

In this article, we predict where expansion of new oil palm plantations is likely to occur in Kalimantan between 2010 – 2020. We do so with a logistic regression model that uses data on recent expansion of oil palm plantations and a set of explanatory variables describing biophysical suitability and infrastructure proximity. We use the regression model to predict expansion and estimate resulting CO_2_ emissions under six alternative land management scenarios, including (i) a BAU scenario, (ii) an alternative BAU scenario in which expansion is constrained to existing permits, and (iii) a moratorium scenario defined by the Ministry of Forestry’s Indicative Moratorium Map [23]. We also evaluate three possible low-emissions land use scenarios: (iv) a peat and forest protection scenario which prohibits the expansion of new plantations on peat lands of any depth and primary and secondary forests; (v) a moderate carbon threshold scenario which prevents plantations on all peat lands and areas with greater than 120 t C ha^**-1**^; and (vi) a strict carbon threshold scenario which precludes plantations from all peat lands and areas with greater than 40 t C ha^**-1**^.

Estimates of *business as usual* GHG emissions due to oil palm expansion in Kalimantan between 2010 and 2020 vary widely. Carlson et al. [16] estimate that 4.4 – 5.5 Gt CO_2_ will be emitted if the more than 9 Mha of existing oil palm permits in Kalimantan are converted. Harris et al. [24] estimate 2.2 Mha of future expansion resulting in 0.9 Gt CO_2_ emissions under a projected BAU scenario. Koh and Ghazoul [25] estimate that BAU expansion would result in 1.0 Gt CO_2_ country-wide over the same time period, without breaking down the estimate by island or province. We expand on these previous analyses by refining predictions of the location of future oil palm plantations, estimating GHG emissions reductions due to recent government policies and industry commitments, and by interpreting results in terms of not only GHG emissions reductions but also the cost-effectiveness of achieving those reductions.

## Methods

### Conceptual Framework

We used a logistic regression model to estimate the probability that land will be converted to an oil palm plantation. Logistic regression relates a binary response variable (in this case, 1 = plantation, 0 = other land use) to a set of explanatory variables. These models have been successfully used to predict land conversion for agricultural expansion across the humid tropics [26–28].

Expected profits, as determined by yields, prices, costs, and risks, drive oil palm expansion. Biophysical factors, including climate, topography and soil characteristics, influence yields.[29]. The international price of palm oil is common across countries, but within countries it varies by distance from ports and other export points. Costs are dependent on biophysical factors, the local labor market, infrastructure (which can be proxied by direct measures, like distance to major roads, or indirect ones, like distance to existing plantations and concessions), and remoteness, which makes inputs more expensive. Cost of capital also contributes to total cost, but interest rates are not a factor that varies across space at a given point in time. Finally, one of the most important components of risk is related to governance. Factors such as corruption and governmental instability can either accelerate or decelerate natural resource exploitation during the investment and production stages of resource-development projects [30,31]. In addition to affecting expected profits directly, prices, costs, and risks also influence profits indirectly via yields, through their impacts on plantations’ use of inputs (e.g., fertilizer) and investments in land-management practices (e.g., terracing).

Given this context, we selected explanatory variables that influence yields, prices, costs, and risks. These variables fall into two broad categories: those describing biophysical suitability, and those describing proximity to infrastructure. We also included district-level binary variables (“dummies”) to control for fixed effects (unobserved sources of mean differences) between districts, which implicitly include governance and labor availability. The risk of overfitting from including these dummies is negligible because the number of dummies (51) is very small compared to the number of degrees of freedom in the model (more than 5,000).

### Model of oil palm plantation expansion

Using the binary response variable on new oil palm plantations as the dependent variable, we estimated generalized linear models with a binomial link function using the GLM function in R v. 2.13.1 [32]. We estimated one model for the entire 2000 – 2010 period and two sub-decade models for 2000 – 2005 and 2005–2010. To generate observations to fit these models, we drew a random sample of 250x250 meter pixels across Kalimantan at elevations less than 1000 meters, above which land is not suitable for oil palm cultivation. Including pixels above this elevation would have artificially inflated the model’s fit by including areas that are far outside the suitable range for expansion [33]. To minimize spatial dependence among observations, we required at least seven kilometers between draws, corresponding to the square root of the average plantation size. The resulting sample sizes were N = 5155 pixels for the 2000 – 2010 model (1022 inside plantations, 4133 outside), N = 2394 pixels for the 2000–2005 model (422 inside, 1972 outside), and N = 2761 pixels for the 2005 – 2010 model (600 inside, 2161 outside). Validation of the model was performed by comparing model predictions to 8 million pixels withheld from the 2000 – 2010 model (0.3 million inside, 7.7 million outside, described in more detail in section 3.1).

Biophysical explanatory variables in the models include elevation and slope [34], annual precipitation, annual dry season precipitation, annual mean temperature [35], soil depth, drainage and acidity [36], and vegetation biomass [37]. Infrastructure explanatory variables include distance to existing oil palm plantations [15], distance to existing oil palm concessions [38], and distance to ports, roads and rivers [39]. We included only major roads, defined as roads longer than 5 km, and the major ports of Pontianak, Sampit, Banjarmasin, Balikpapan, and Samarinda. Distance to minor roads and palm oil mills were omitted in order to avoid simultaneity bias, as roads and mills are frequently jointly determined with plantation expansion [40]. All variables were defined at the beginning of the time period analyzed (i.e., 2000 for analyses of plantation expansion during 2000–2010 and 2000–2005). Distance to existing plantations was defined at the beginning of 2005 for analysis of expansion during 2005–2010. All data were processed at 250-meter resolution and are available online at: www.wri.org/applications/maps/suitability-mapper/.

We used robust Huber-White standard errors clustered by district to test the statistical significance of these variables. Clustered robust standard errors correct for heteroskedasticity and spatial correlation of the error term [41,42]. Failure to account for these problems can result in severely underestimated standard errors and exaggerated indications of statistical significance [43]. The number of districts in the sample (N = 52) was sufficiently large to satisfy the asymptotic requirements of the clustering adjustment [43].

### Model statistics and robustness checks

To reduce the risk of omitted variables bias, in which coefficients on variables included in the model are biased as a result of picking up the effects of excluded variables [40], we included the full set of explanatory variables in our preferred model, regardless of their significance levels. Omitted variables bias is a more serious problem in multivariate models than multicollinearity, which inflates the standard errors of coefficients but does not bias the coefficients themselves. However, we did investigate the effects of multicollinearity on the significance of the coefficients and the impacts on the model of excluding variables that showed evidence of multicollinearity (see Supplementary Information). We began by identifying relatively large (> 0.7) pairwise correlations between the explanatory variables. We found four such correlations, involving five variables: distance to oil palm concessions and distance to existing oil palm plantations, distance to oil palm concessions and elevation, slope and elevation, and mean temperature and elevation (S1 Table). Despite these correlations, three of the variables were highly significant in the full model (P < 0.001), and excluding the two that were not (slope and mean temperature; P > 0.7) did not substantially change their coefficients or standard errors.

We also examined variance inflation factors (VIF; S2 Table), which provide a better indicator of multicollinearity because they account for linear dependence within the full set of other explanatory variables, not just pairs of variables. All the VIFs were much less than 10, a common threshold for detecting multicollinearity, and all but three were less than 2.5, indicating little evidence of large correlations in a multivariate sense [44]. In addition, despite having the 2nd, 3rd, and 4th largest VIFs, distance to oil palm plantations, elevation, and distance to major ports all had P values < 0.001 in the full model, indicating that multicollinearity did not substantially inflate their standard errors.

As a final check, we specified a parsimonious model by iteratively removing the least significant explanatory variable until all remaining variables were significant at P < 0.05. The result was a model that included the same set of highly significant variables as the full model, with coefficients that were not substantially different between the two models (S3 Table). Using an information criterion such as the AIC or BIC as the stopping rule for this backwards selection procedure yielded the same final parsimonious model. Basing the stopping rule on P values is more appropriate, however, as the AIC and BIC are not valid measures of model fit when data are clustered [45]. We use this model to predict GHG emissions under each scenario, in order to test whether our results are sensitive to model specification (presented in section 3.3).

### Scenario development

Gunarso et al. [15] estimated an increase of 350,000 ha of oil palm plantations between 2000 – 2005 and 1,800,000 ha between 2006 – 2010, totaling 2.2 Mha for the decade. Due to the large difference between the expansion rates observed during the first and second half of the decade, we used two estimates for the magnitude of expansion. The first scenario is based on applying the decade total of 2.2 million hectares (Mha) to the 2010 – 2020 period. The second scenario is based on the assumption that the observed expansion rate of 1.8 Mha in the second half of the past decade will continue, resulting in 3.6 Mha of expansion during 2010 – 2020. This range brackets the 2.9 Mha required to double the observed 2010 oil palm plantation extent.

We modeled future plantation expansion by assigning projected expansion (either 2.2 or 3.6 Mha) within the study area, with the probability of expansion at a given pixel determined by the prediction of the logistic regression model. We required expansion to occur in clusters averaging 49 km^2^, corresponding to the mean area of observed plantations, in order to better represent realistic expansion patterns across the landscape. To estimate precision of the resulting estimates, we conducted 100 bootstrapped replicates for each expansion scenario.

**Fig 1:**
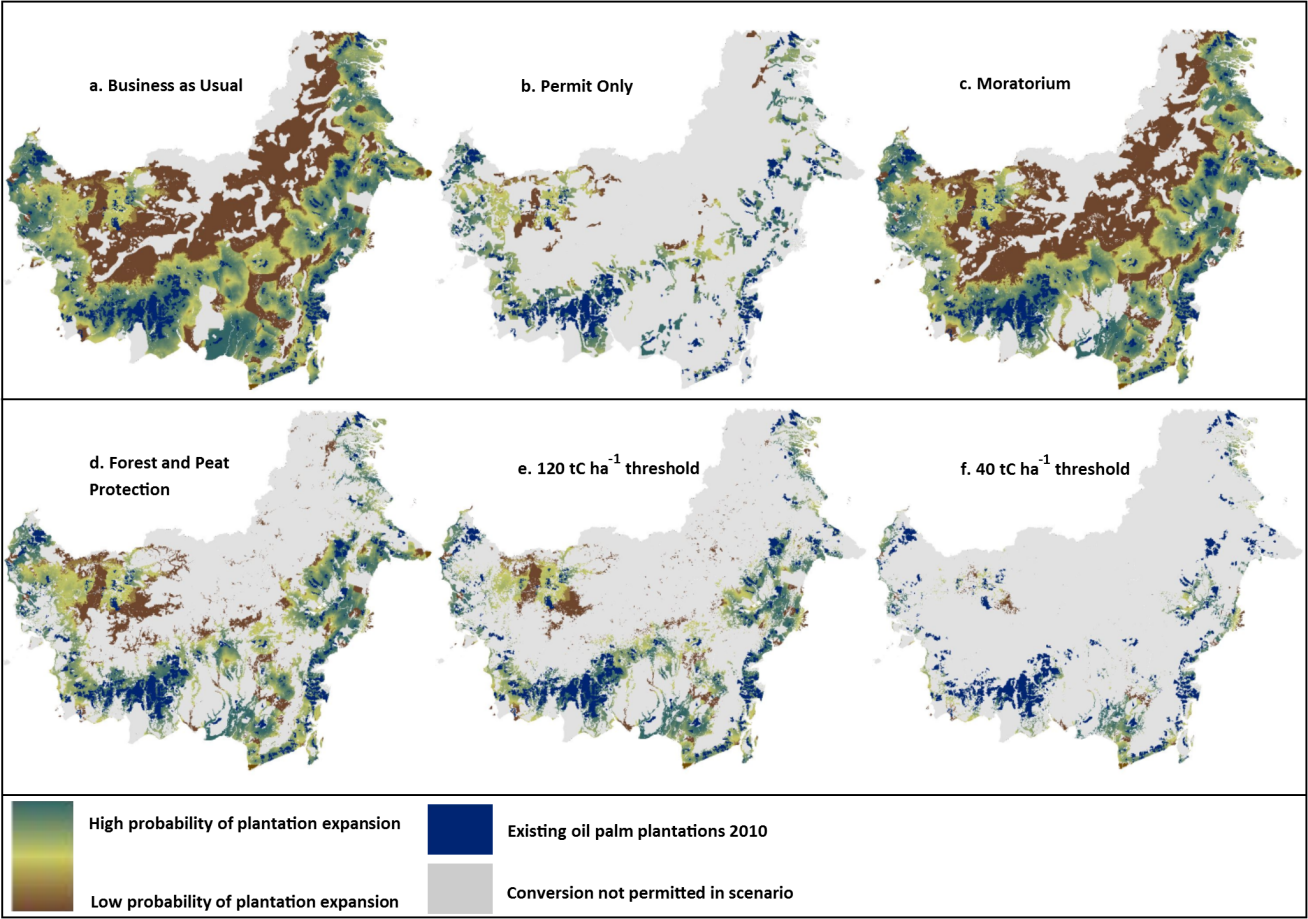
Probability of Oil Palm Plantation Expansion in Kalimantan under six alternative expansion scenarios.

We generated 12 predictions of the locations of new plantations and associated CO_2_ emissions according to six alternative policy scenarios using a prediction for each of the lower (2.2 Mha) and higher (3.6) aggregate area of new plantations. To develop the alternative expansion scenarios we used maps of protected areas, settlements, legal land classifications, and the moratorium boundaries from the Indonesian Ministry of Forestry [23,46], and peat lands from Wetlands International [47]. Each scenario is described here, and shown in Fig 1:

- Business as Usual (BAU) – Excludes existing oil palm plantations, settlements, and areas that qualify for protection under Indonesian law [48]. In the BAU scenario we did not constrain future expansion to permitted areas but found that approximately 40% of future plantation expansion occurs on land already licensed for oil palm, but not yet converted. Conversely this suggests that 60% of expansion will occur in new permits in areas that are expected to be more attractive for oil palm expansion than within existing permits.
- Permit constrained – In the permit constrained scenario we limit expansion to areas within existing permits, including *Hak Guna Usaha* (HGU) permits, and initial permits for plantation planning (e.g. *izin prinsip* and *izin lokasi*) [38]. This data set may not be complete if permits agreed to at the district level are not registered by Indonesia’s Ministry of Forestry. According to this dataset there are 8.6 Mha of permits designated for oil palm expansion in Kalimantan which have not yet been converted to plantations.
- Moratorium—Excludes existing oil palm plantations, settlements, protected area, and areas protected from new permits by the moratorium. Although multiple versions of the moratorium map exist, as the Ministry of Forestry is required to update the map semi-annually, we use the map provided November 2013 [23].
- Peat and forest protection – Excludes existing oil palm plantations, settlements, protected areas, peat lands of any depth [47], and primary and secondary forests [46]. This scenario most closely resembles the scenario in which all companies commit to a zero-deforestation palm oil pledge, assuming that the adopted definition of zero-deforestation prevents conversion of both primary and secondary forests.
- Moderate carbon threshold—Excludes existing oil palm plantations, settlements, areas that qualify for protection under Indonesian law, peat lands of any depth, and areas with above- and below-ground biomass carbon stock values greater than 120 tC ha^-1^. We selected a threshold of 120 tC ha^-1^ to exclude intact and non-degraded forests in insular Asia [49].
- Strict carbon threshold—Excludes existing oil palm plantations, settlements, areas that qualify for protection under Indonesian law, peat lands of any depth, and areas with above- and below-ground biomass carbon stock values greater than 40 tC ha^-1^. We selected a threshold of 40 tC ha^-1^ to reflect the time-averaged carbon stock of oil palm plantations and the provisional definition of high carbon stock proposed by the Roundtable on Sustainable Palm Oil [50,51]. There is insufficient area to accommodate all of the projected expansion that have less than 40 tC ha^-1^ and greater than 0.5 likelihood of expansion estimated by the regression model. We therefore allowed expansion in areas with up to 80 tC ha^-1^ to accommodate 3.6 Mha expansion and up to 50 tC ha^-1^ to accommodate the 2.2 Mha expansion.

### CO_2_ emissions estimation

We estimated CO_2_ emissions for each scenario by summing the above- and below-ground biomass carbon and peat soil carbon estimates of each pixel within the projected expansion area. We used carbon stock data from Saatchi et al. [37], who derived their estimates from satellite observations calibrated with field measurements. We used their estimates of uncertainty determined by comparing field measurements withheld from their model with the corresponding model result, to characterize the confidence of our results. We converted C to CO_2_ using a ratio of 44/12, and assumed all biomass carbon stocks are emitted in the year of conversion. Additionally, we used carbon stock data from Baccini et al. [52] to test the sensitivity of this approach to the input carbon stock dataset.

We estimated CO_2_ emissions from peat conversion using a 30-year time-averaged emission factor of 99 tCO_2_ ha^-1^ yr^-1^ ± 37 tCO_2_ ha^-1^ yr^-1^ over the projection period [53]. This emission factor takes into account emissions due to oxidative decomposition but not due to peat fires or due to off-site impacts of drainage [16,54]. Nor does it include methane or nitrous oxide emissions, as these are relatively small and fall well within the uncertainty bounds [55]. We calculated net emissions by subtracting the time-averaged oil palm plantation carbon stock estimate of 147 tCO_2_ ha^-1^, from gross CO_2_ emissions estimates [50].

### Scenario Evaluation

We assessed the potential mitigation that would be offset by leakage by estimating the area currently in agricultural use within each expansion scenario. Leakage in this context is the displacement of forest and peat land conversion activities and their resulting GHG emissions from within to outside the projected oil palm expansion area. Other studies suggest that emissions reduction activities that limit the supply of goods and services that people depend on for their livelihoods can cause these activities to shift elsewhere, potentially undermining the effectiveness of the mitigation strategy [56]. We estimated leakage by quantifying the area currently under agricultural production according to the Ministry of Forestry [46] within each oil palm expansion scenario.

Additionally, information on the potential impacts on profits can provide insight into the political feasibility of different policy scenarios. Scenarios that are equivalent in terms of the amount of plantation expansion that they allow (in our scenarios, either 2.2 MHa or 3.6 Mha) are not equivalent to the industry and the industry’s political supporters if their impacts on profits differ. We assessed the impact of a particular scenario on the profitability to the palm oil industry by calculating the difference between the mean propensity score for that scenario and the mean score for the corresponding BAU scenario. Pixels with identical propensity scores are ones that the industry views as equally profitable for plantations, based on the observed characteristics included in the logistic regression model. If the mean propensity score decreases relative to the BAU scenario, then mean oil palm profits decrease as well. Although mean propensity scores can be interpreted as ordinal measures of profitability, they are not necessarily cardinal measures. For example, a 10% reduction in mean score compared to the mean score for the BAU scenario implies a reduction in profit, but not one that necessarily equals 10%.

Information on impacts on profits is also useful because it can be used to determine the cost-effectiveness of the scenarios for reducing GHG emissions, given that the industry’s forgone profits represent the costs of achieving the reductions. We calculated a proxy for the cost-effectiveness of the emissions reductions achieved by a given scenario by comparing the difference in GHG emissions between that scenario and the BAU scenario to the corresponding difference in the mean propensity scores.

## Results

### Model of oil palm plantation expansion

Table 1 shows the logistic regression model based on 2000 – 2010 data predicting likelihood of oil palm plantation expansion. P values are small (P < 0.05) for most of the infrastructure variables, indicating significant negative effects of distance to existing concessions, existing plantations, major ports and major roads on plantation expansion. This finding supports previous work by Gaveau et al. [57], who found that oil palm concessions are commonly located in easy to access and non-remote areas.

**Table 1:** Probability of oil palm plantation expansion: logistic regression model.

	Coefficient	Standard Error	Pr(>|z|)
**Intercept**	-1.374	1.626	0.398
**Distance to existing oil palm concessions (km)**	-1.146	0.292	0.000*
**Distance to existing oil palm plantations (km)**	-0.391	0.090	0.000*
**Distance to major roads (km)**	-0.544	0.306	0.076
**Distance to rivers (km)**	0.198	0.190	0.299
**Distance to major ports (km)**	-0.732	0.131	0.000*
**Elevation (m)**	-0.089	0.024	0.000*
**Slope (%)**	-0.005	0.020	0.807
**Annual Rainfall (10 cm)**	-0.008	0.017	0.620
**Rainfall in the Driest Quarter (10 cm)**	0.035	0.176	0.844
**Annual Mean Temperature (°C)**	0.016	0.055	0.770
**Soil Depth (cm)**	0.005	0.023	0.812
**Soil Acidity (pH)**	-0.073	0.112	0.517
**Soil Drainage Index**	0.213	0.100	0.033*
**Biomass Carbon Stocks (10 T C / ha)**	-0.016	0.021	0.439

The model also included 51 district-level binary factors (not shown). Total number of observations: 5,155. Reported standard errors are Huber-White robust estimates, clustered by district. P-values are based on a two-sided z-test of the null hypothesis that the parameter estimate equals zero. Asterisks indicate significance at P < 0.05.

P values for the biophysical variables, with the exception of elevation and soil drainage, are large, indicating little evidence of significant effects. As discussed earlier, the insignificance of slope and mean temperature might be due to multicollinearity. Limited variation across pixels within the same district could also be responsible for the insignificance of some of the biophysical variables: the inclusion of the district-level dummy variables strips out the between-district variation in all the explanatory variables in the model.

The signs, magnitudes, and significance levels of the coefficients on the explanatory variables do not differ substantially between the model based on 2000 – 2010 data and the two models based on 2000–2005 data and 2005 – 2010 data (S4 Table). However the coefficients on the district binary factors are not as static as the rest of the explanatory variables in the model (S1 Fig). We found that coefficients change significantly (P < 0.05) on thirteen district binary factors in West, Central and South Kalimantan [58]. All thirteen cases correspond to districts with relatively little oil palm prior to 2005, and in each case the district binary factor coefficient changed from significantly negative in the 2000 – 2005 period to not significantly different from zero in the 2005 – 2010 period (S2 Fig). This shift indicates that there are unobserved changes in the political economy of these districts which encouraged new oil palm expansion in the second half of the decade. This finding reveals the importance of developing a better understanding of underlying political processes to improve prediction of future land use change.

To evaluate model predictions we used a Relative Operation Characteristics (ROC) curve to identify a threshold probability value that maximizes true positives while minimizing false positives [59]. We applied the resulting threshold to create a binary prediction of ‘probable’ versus ‘not-probable’, which we then used to assess model accuracy against observed oil palm plantations in 2010, withholding the 5155 pixels used to create the model [60]. Overall accuracy of the final model for 2000–2010 is 78.6%, with a false positive rate of 22.2% and a false negative rate of 6.1%. Importantly, false positives in this context correspond to areas where expansion is predicted by the model but not observed. This type of misclassification is to be expected in the case where there is more land available for oil palm plantation development than is currently occupied by plantations.

### Prediction of *CO*_*2*_ *emissions under alternative scenarios*

We estimate that under a BAU scenario 128.4 ± 43.8 Mt CO_2_ yr^-1^ will be emitted if 2.2 Mha of oil palm is developed over the decade, and 211.4 ± 71.9 Mt CO_2_ yr^-1^ will be emitted if 3.6 Mha of oil palm is developed (Table 2). When expansion is constrained to existing permits the area available for expansion is just 8.3 Mha, compared to 38.4 Mha under the BAU scenario, but emissions stay within ± 3 Mt CO_2_ yr^-1^ of the BAU scenario. In the permit constrained scenario, emissions decrease slightly relative to BAU when 2.2 Mha of oil palm is developed, to 125.1 ± 43.4 Mt CO_2_ yr^-1^, and increase slightly when 3.6 Mha of oil palm is developed, to 212.8 ± 72.1 Mt CO_2_ yr^-1^.

**Table 2:** Estimated net CO_2_ emissions due to oil palm plantation establishment from 2010 – 2020.

	Biomass CO_2_ emissions (Mt CO_2_ yr^-1^)	Peat CO_2_ emissions (Mt CO_2_ yr^-1^)	Total CO_2_ emissions (Mt CO_2_ yr^-1^)	CO_2_ emissions relative to BAU
**2.2 Mha Expansion**				
**Business as Usual**	87.8	40.7	128.4 ± 43.8	—
**Permit constrained**	92.5	32.7	125.1 ± 43.4	-2.6%
**Moratorium**	83.5	32.5	116.0 ± 42.5	-9.7%
**Peat and Forest Protection**	83.1	0	83.1± 38.1	-35.3%
**120 tC ha** ^**-1**^ **threshold**	69.8	0	69.8 ± 36.7	-45.6%
**40 tC ha** ^**-1**^ **threshold**	51.8	0	51.8 ± 33.8	-59.6%
**3.6 Mha Expansion**				
**Business as Usual**	145.0	66.4	211.4 ± 71.9	—
**Permit constrained**	154.7	58.1	212.8 ± 72.1	0.6%
**Moratorium**	146.0	46.6	192.6 ± 69.4	-8.9%
**Peat and Forest Protection**	137.1	0	137.1 ± 62.4	-35.2%
**120 tC ha** ^**-1**^ **threshold**	115.3	0	115.3 ± 60.1	-45.5%
**40 tC ha** ^**-1**^ **threshold**	95.1	0	95.1 ± 57.2	-55.0%

Two scenarios of overall expansion, 2.2 Mha and 3.6 Mha, and six scenarios of expansion trajectories are presented. Uncertainty estimates are presented after ± sign, and include uncertainty associated with input carbon stock estimates and 95% confidence intervals derived from bootstrapping.

By limiting expansion under the moratorium, the peat and forest protection scenario, the moderate carbon threshold, and the strict carbon threshold, the area available for expansion is reduced to 35.5 Mha, 18.1 Mha, 12.1 Mha, and 3.4 Mha, respectively, compared to 38.4 Mha under the unconstrained BAU scenario. The average carbon stock values within each scenario likewise drop from 149 tC ha^-1^ under the moratorium policy to 30 tC ha^-1^under the strict carbon threshold scenario, compared to 179 tC ha^-1^ under BAU.

We find that by limiting new plantations under the current and proposed policy scenarios, shown in Fig 1, emissions from oil palm expansion in Kalimantan could be reduced by between 8.9% and 59.6% relative to BAU. We estimate that the moratorium, which does not allow new permits in primary forests and peat lands, is reducing emissions from oil palm expansion by 8.9 – 9.7% annually (12.4– 18.8 Mt CO_2_ yr^-1^). By allowing expansion into areas where permits have not yet been granted the moratorium scenario results in lower GHG emissions than the permit constrained scenario, which allows expansion in existing permits only, many of which contain forests with relatively high carbon stocks.

There is greater uncertainty around the potential GHG emissions avoided by implementing a low-emissions land use strategy, as the definition of ‘low carbon’ or ‘degraded’ has not been established in Indonesian law [61]. Preventing expansion onto peat lands and into primary and secondary forests could reduce emissions by 35.2 – 35.3% (45.3 – 74.3 Mt CO_2_ yr^-1^) relative to BAU. The additional emissions reduction achievable by this scenario is due to the protection of all peat lands and secondary forests, including those within existing permits, which are exempted the current moratorium. The 120 tC ha^-1^ threshold scenario could reduce emissions by 45.5 – 45.6% (58.6 to 96.1 Mt CO_2_ yr^-1^) and the 40 tC ha^-1^ threshold scenario could reduce emissions by 55.0 – 59.6% (76.6 and 116.3 Mt CO_2_ yr^-1^) relative to BAU. CO_2_ emissions predicted under each scenario, and the estimated reduction in CO_2_ emissions compared to BAU, are presented in Table 2.

### Robustness checks

To evaluate model robustness we compared the results based on the 2000 – 2010 model with the results based on the 2005 – 2010 model. Using the latter model we estimated expansion in two five-year phases, with half of expansion occurring from 2010 – 2015 and the rest from 2015 – 2020. For the first half of the decade the model estimates probability of oil palm expansion using distance to existing plantations in 2010 as an input. In the second half of the decade the same model is used but distance to the modeled location of plantations in 2015 is substituted. We found that GHG emissions from oil palm expansion averaged just 2% higher using the 2005 – 2010 model than the results using the 2000 – 2010 model.

We also compared the GHG emissions estimates in each scenario using the full model parameterized with 14 explanatory variables (Table 1), to the parsimonious model parameterized with 5 explanatory variables (S3 Table). We found that GHG estimates using the parsimonious model ranged from 4.5 Mt CO2 lower to 12.6 Mt higher, or -3.8% lower to 11.6% higher, and averaged 2% higher than the full model presented in Table 1. These are not large differences and the estimates fall well within the confidence intervals presented in Table 2.

Finally, we compared results using our model with Saatchi et al. (2011) biomass data and with Baccini et al. (2012) biomass data. We found that the resulting emissions estimates using the Baccini et al. data averaged 6% lower than the results using the Saatchi et al. data. This may be due to the difference in time between these datasets; Saatchi et al.’s map represents carbon stocks in the year 2000 while Baccini et al.’s map represents carbon stocks in 2007, during a period when significant deforestation occurred in Kalimantan [8].

### Assessment of leakage

We found that under the BAU scenario and the permit constrained scenario between 39% and 45% of the area of projected oil palm expansion over the decade occurs on land that is already cultivated. The proportion of expansion on cultivated land drops slightly relative to BAU under the moratorium scenario, to 39%- 41%. However under the low-emissions expansion scenarios including peat and forest protection and moderate and strict carbon thresholds the proportion of expansion occurring on cultivated land increases relative to BAU to 47%- 54%.

These results indicate that under the low-emissions expansion scenarios there would be between 0.04 and 0.39 Mha of additional agriculture land impacted by oil palm expansion that may shift elsewhere. Additional research is needed to understand the type of agricultural activities in these areas, the likelihood that these activities would shift elsewhere, whether the shift would occur within the region or beyond regional/national borders, and the GHG emissions impacts of this shift.

### Assessment of impacts on industry profitability

We estimate that the average propensity score of pixels selected for expansion drops by 4–15% below business as usual across the assessed scenarios (Table 3). In the 2.2 Mha expansion scenario the average propensity score is 0.80 under BAU, but this drops by 12% to 0.71 under the strictest carbon threshold scenario. Likewise in the 3.6 Mha expansion scenario BAU the average propensity score is 0.74 under BAU, which drops by 15% to 0.63 under the strictest carbon threshold. These results suggest relatively moderate reductions in profits, although as noted earlier the propensity scores capture the effects of only observed factors that affect profits and are ordinal, not cardinal, measures of impacts on profits. The scenario with the smallest change relative to BAU is the peat and forest protection scenario, in which the average propensity score decreases by 4% relative to BAU in both the 2.2 Mha and 3.6 Mha cases. These results suggest that the oil palm industry would be most supportive of the peat and forest protection scenario and least supportive of the strictest carbon threshold.

**Table 3:** Average propensity scores within areas of predicted expansion.

	Average CO_2_ emissions (Mt CO_2_ ha^-1^ yr^-1^)	Average propensity score	Average propensity score change relative to BAU	Ratio of change in average propensity score to change in emissions (1000 t CO_2_ ha^-1^ yr^-1^)
**2.2 Mha expansion**				
**Business as Usual**	58.36	0.80 ± 0.13	—	—
**Permit constrained**	56.86	0.75 ± 0.09	-7%	36.087
**Moratorium**	52.73	0.74 ± 0.12	-8%	11.362
**Peat and Forest Protection**	37.77	0.77 ± 0.11	-4%	1.749
**120 tC ha** ^-**1**^ **threshold**	31.73	0.73 ± 0.10	-10%	2.891
**40 tC ha** ^-1^ **threshold**	23.55	0.71 ± 0.08	-12%	2.844
**3.6 Mha expansion**				
**Business as Usual**	58.72	0.74 ± 0.12	—	—
**Permit constrained**	59.11	0.69 ± 0.09	-7%	-130.398 *
**Moratorium**	53.50	0.70 ± 0.11	-5%	6.897
**Peat and Forest Protection**	38.08	0.71 ± 0.09	-4%	1.405
**120 tC ha** ^-**1**^ **threshold**	32.03	0.68 ± 0.08	-8%	2.135
**40 tC ha** ^-1^ **threshold**	26.42	0.63 ± 0.07	-15%	3.312

The ratio of change in average propensity score to emissions reductions is calculated as the difference between the average propensity score relative to the BAU scenario divided by the difference between the average CO_2_ emissions relative to the BAU scenario.

* Negative value results from an estimate increase in GHG emissions in the permit constrained scenario when area increases to 3.6 Mha. Uncertainty estimates include 95% confidence intervals derived from bootstrapping.

The ratio of propensity score loss to emissions reductions provides a proxy for the relative cost-effectiveness of each scenario, with lower ratios indicating lower cost options per ton of avoided emissions (Table 3). The moratorium scenario is the least cost effective of the low-emissions scenarios evaluated, in part because the scenario results in few estimated emissions reductions. The peat and forest protection scenario has the lowest unit-cost emissions reductions of the scenarios evaluated. Hence, in addition to being favored by industry, it represents the least costly way to achieve emissions reductions. Among the carbon threshold scenarios the strict carbon threshold is slightly more cost effective than the moderate threshold in the 2.2 Mha expansion case, but less cost effective in the 3.6 Mha case.

This assessment reveals a decrease in profitability of the oil palm industry if plantations are diverted onto low carbon stock land. However, we are not able to discern whether this reduction is due to lower yields, lower prices, or higher costs. It is clear that one or more of these negative effects would occur, but additional data and research is needed to disaggregate these effects.

## Discussion

Throughout the tropics agriculture is a principal driver of deforestation [62], which contributes 7–14% of global GHG emissions [63]. As agricultural expansion is expected to continue through 2050, more efficient patterns of land use will be needed to reconcile this growth with the goals of forest protection and climate change mitigation. The results of this study suggest that diverting oil palm expansion in Kalimantan away from high carbon stock landscapes could allow the region both to meet oil palm area-expansion goals and avoid significant GHG emissions. However, we estimate that achieving the industry’s proportional contribution to the nation-wide GHG goal of reducing emissions by 26–41% by 2020 will require more stringent restrictions on expansion than have previously been put in place at the national level, and these restrictions will reduce industry profits, which can be expected to generate resistance to those restrictions.

### Impact of the Moratorium

Indonesia’s moratorium has been criticized for not protecting secondary forests, for exempting more than 3.5 Mha of permits in high carbon stock forests and peat lands, and for leaving loopholes for food and energy security, all of which reduce its effectiveness of avoiding GHG emissions [64]. Previous research has shown that only a quarter of the area protected by the moratorium benefits from additional legal protection beyond that already provided by protected area designations [65]. Additionally, Sloane et al. [66] demonstrate that the areas protected by the moratorium are more remote and therefore less likely to be converted.

We estimate that the moratorium reduces emissions from oil palm expansion by approximately 9% below BAU, or less than half of the emissions reductions needed for the palm oil industry to contribute it proportional share of the nation-wide mitigation goal of 26%. However, it is worth noting that the moratorium was not designed to reduce emissions on its own. Rather, its primary intent is to reform governance of forest resources and improve forest management in order to reduce GHG emissions in the long run [64]. For a policy that was not designed for the express purpose of emissions mitigation, it does make limited progress towards this goal. The government has extended the original moratorium, which now applies through May of 2015 [67]. Our analysis suggests that extending the moratorium through 2020 is a productive mitigation strategy, but may not be as cost effective as other land management policies aimed at GHG mitigation in the oil palm sector.

### Impact of a Low-Emissions Land Use Strategy

Diverting agricultural expansion to low carbon stock landscapes is a mitigation option included in Indonesia’s National REDD+ Strategy and in provincial low-emissions development plans [11,19,20]. However, specific definitions of low carbon stock have not yet been agreed upon. In this study we evaluated alternative formulations of a low-emissions oil palm expansion strategy in order compare scenarios based on their expected GHG impacts.

We find that preventing oil palm expansion on all peat lands of any depth, including those within previously permitted but unconverted concessions, and all forest land including secondary forests, could significantly increase the GHG emissions reductions achievable from the moratorium, to 35% below BAU. Restricting oil palm expansion to areas without peat and with less than 120 tC ha^-1^ or 40 t C ha^-1^ will achieve reductions of 45% and 55–60% below BAU, respectively. Though significant uncertainty remains, these findings suggest that the land management strategies evaluated in this study could achieve or even exceed the palm oil industry’s proportional share of the national emissions reduction goal. The peat and forest protection scenario looks especially attractive: it has the lowest impact on industry profits, while still resulting in emissions reductions of 35% below BAU, and as a result of these two effects achieves the most cost-effective emissions reductions.

### Limitations to Implementation

While our study demonstrates that policies which constrain where oil palm cultivation may occur have the potential to avoid significant GHG emissions in Kalimantan, the question of whether these policies can be fully implemented and enforced remains. There are governance, institutional, and economic factors that may prevent land use planners and decision-makers from fully implementing more stringent restrictions on where oil palm cultivation may expand [67]. Changes in the coefficients on the district-level dummy variables in our model between 2000– 5 and 2005–10 indicate that these factors are not fixed and that changes in them can have important impacts on plantation expansion.

The regulatory regime responsible for managing oil palm expansion in Indonesia has proven ineffective at adequately implementing and enforcing pollution mitigation strategies in the sector in the past, indicating that the potential mitigation policies may be weakly applied in the future [68]. While strong regulations may be developed at the national or provincial level, decentralized government agencies are responsible for ensuring compliance with these regulations at the district level. These agencies are often understaffed and under resourced, and thus their primary interest may lie in supporting development [68]. Even when incentives are not aligned with industry, district agencies frequently do not have access to data on which to assess permit applications for their potential negative environmental impacts, including accurate maps of the moratorium, peat land extent, and locally specific carbon stock data [65,69].

In addition, restricting oil palm expansion to low carbon stock, non-forest land may be limited by conflicts with local communities. We find that at least 40 – 50% of the area of predicted expansion occurs on lands already under some form of cultivation, indicating significant occupation of these areas. Previous studies have observed conflicts between palm oil companies and local communities, particularly in cases where land rights are unclear, benefits are unevenly distributed, and in the absence of free, prior, and informed consent of local communities [70,71]. Additional research which takes into account customary land tenure rights is essential to more accurately estimate the potential GHG emissions reductions achievable in the sector.

Due to inadequate enforcement or incentive mechanisms to ensure compliance with regulations, industry buy-in is essential to the success of any long term mitigation strategy in the sector [72]. However the potential decrease in profitability of the oil palm industry if plantations are diverted onto low carbon stock land poses an obstacle to strong industry support. Experience with the implementation of the moratorium, in which there was pushback from palm oil companies who perceived this environmental regulation as a threat to job creation and economic growth, provides an example of the challenges to design and implementation of low-emissions plans in the sector [64]. While palm oil producers may be averse to internalizing the costs of shifting to a low-emissions expansion scenario, the new wave of corporate commitments to eliminate deforestation and peat land conversion from the palm oil supply chain indicates that industry commitment to sustainable palm oil production is growing.

The palm oil industry in Indonesia has attracted attention for its economic benefits, as well as its environmental impacts. This study estimates the potential for the oil palm sector in Kalimantan to achieve GHG emissions reductions by strengthening policies which delineate where expansion may occur. We estimate that emissions in Kalimantan can be reduced by 9–10% by extending the current moratorium on new concessions in primary forests and peat lands, 35% by limiting expansion on all peat and forestlands, 46% by limiting expansion to areas with moderate carbon stocks, and 55–60% by limiting expansion to areas with low carbon stocks. We conclude that growth of the oil palm sector can be consistent with the Indonesia’s climate change mitigation goal, as long as a carefully designed expansion plan is implemented.

## Supporting Information

S1 Fig Comparison of district binary factor coefficients between sub-decade models. (DOCX)

S2 Fig Districts in which binary factor coefficients shift from significantly negative to non-significantly different from zero between the first and second half of the 2000 – 2010 decade. (DOCX)

S1 Table Pairwise correlations between explanatory variables included in the full model. (DOCX)

S2 Table Variance inflation factors (VIF). (DOCX)

S3 Table Parsimonious model specified by iteratively removing the least significant explanatory variable until all variables are significant at P < 0.05. (DOCX)

S4 Table Comparison of models predicting oil palm plantation expansion based on 2000 – 2005 data and 2005 – 2010 data. (DOCX)
