# Supplementary material for: Reconciling Oil Palm Expansion and Climate Change Mitigation in Kalimantan, Indonesia
Source: PLoS One. 2015 May 26;10(5):e0127963. doi: 10.1371/journal.pone.0127963 (PMC4444018; doi:10.1371/journal.pone.0127963)
Supplement: S1 Fig — (DOCX) [file pone.0127963.s001.docx]

S1 Figure. Comparison of dummy variable coefficients between sub-decade models. Dummy coefficients are plotted in increasing order according to the 2000 – 2005 model. Blue lines bound the area within which coefficients are not statistically different from zero (P > 0.05). Negative values indicate that oil palm was less likely to expand into the district during the period, holding all other factors constant. The coefficients on thirteen districts were significantly different in the second half of the decade – these districts correspond to the thirteen districts in the left side of the chart in which the coefficients shift from statistically different from zero in the 2000 – 2005 period (black diamonds) to not statisticall different in the 2005 – 2010 period (grey squares).
