# Supplementary material for: Reconciling Oil Palm Expansion and Climate Change Mitigation in Kalimantan, Indonesia
Source: PLoS One. 2015 May 26;10(5):e0127963. doi: 10.1371/journal.pone.0127963 (PMC4444018; doi:10.1371/journal.pone.0127963)
Supplement: S2 Fig — (DOCX) [file pone.0127963.s002.docx]

**S2 Figure. Districts in which dummy coefficients shift from significantly negative to non-significantly different from zero between the first and second half of the 2000 – 2010 decade.** Districts in this category include Banjar, Barito Kuala, Tapin, Hulu Sungai Selatan and Tabalong in South Kalimantan, Kapua, Lamandau, Pulang Pisau, Gunung Mas, and Palangka Raya in Central Kalimantan, and Kapuas Hulu, Landak and Sanggau in West Kalimantan. These correspond to areas where expansion of oil palm plantations in the first half of the decade (light blue) was much lower than in the second half of the decade (light green).


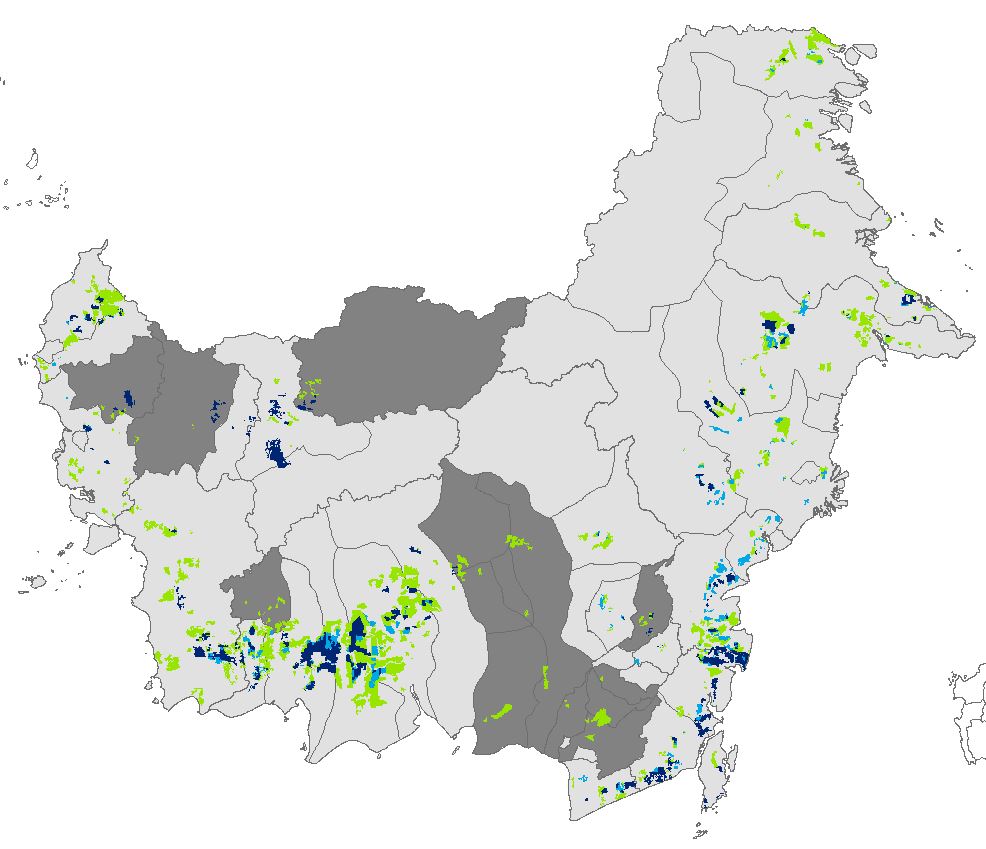

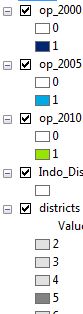

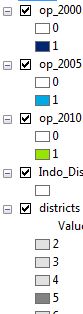

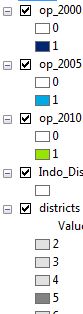

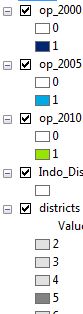


Oil Palm Plantations 2000

Oil Palm Plantations 2005

Oil Palm Plantations 2010

Districts with shifting dummy coefficients
