# Supplementary material for: Reconciling Oil Palm Expansion and Climate Change Mitigation in Kalimantan, Indonesia
Source: PLoS One. 2015 May 26;10(5):e0127963. doi: 10.1371/journal.pone.0127963 (PMC4444018; doi:10.1371/journal.pone.0127963)
Supplement: S1 Table — (DOCX) [file pone.0127963.s003.docx]

**S1 Table. Pairwise correlations between explanatory variables included in the full model.** Correlations > 0.7 are highlighted.

|  | **C Stocks** | **Con-cession dist.** | **Plant-ation dist.** | **Soil Depth** | **Soil Acid-ity** | **Soil Drain-age** | **Road dist.** | **River dist.** | **Slope** | **Elev-ation** | **Dry season rainfall** | **Annual Rainfall** | **Mean Temp** | **Port dist.** |
| --- | --- | --- | --- | --- | --- | --- | --- | --- | --- | --- | --- | --- | --- | --- |
| **C Stocks** | -- | 0.35 | 0.44 | -0.35 | 0.18 | 0.33 | 0.20 | 0.00 | 0.44 | 0.39 | 0.36 | 0.06 | -0.29 | 0.16 |
| **Concession dist.** | 0.35 | -- | **0.77** | -0.32 | 0.16 | 0.19 | 0.64 | 0.24 | 0.50 | **0.73** | 0.46 | 0.11 | -0.61 | 0.14 |
| **Plantation dist** | 0.44 | 0.77 | -- | -0.28 | 0.17 | 0.14 | 0.56 | 0.12 | 0.48 | 0.62 | 0.49 | 0.20 | -0.52 | 0.15 |
| **Soil Depth** | -0.35 | -0.32 | -0.28 | -- | 0.09 | -0.35 | -0.17 | -0.10 | -0.44 | -0.47 | -0.20 | -0.02 | 0.36 | -0.11 |
| **Soil Acidity** | 0.18 | 0.16 | 0.17 | 0.09 | -- | 0.09 | 0.14 | 0.07 | 0.20 | 0.21 | -0.02 | -0.02 | -0.23 | 0.10 |
| **Soil Drainage** | 0.33 | 0.19 | 0.14 | -0.35 | 0.09 | -- | 0.06 | 0.04 | 0.33 | 0.33 | 0.18 | 0.05 | -0.24 | 0.12 |
| **Road dist.** | 0.20 | 0.64 | 0.56 | -0.17 | 0.14 | 0.06 | -- | 0.09 | 0.41 | 0.58 | 0.32 | 0.07 | -0.48 | 0.12 |
| **River dist.** | 0.00 | 0.24 | 0.12 | -0.10 | 0.07 | 0.04 | 0.09 | -- | 0.12 | 0.24 | 0.00 | 0.00 | -0.22 | 0.10 |
| **Slope** | 0.44 | 0.50 | 0.48 | -0.44 | 0.20 | 0.33 | 0.41 | 0.12 | -- | **0.71** | 0.33 | 0.06 | -0.56 | 0.18 |
| **Elevation** | 0.39 | 0.73 | 0.62 | -0.47 | 0.21 | 0.33 | 0.58 | 0.24 | 0.71 | -- | 0.40 | 0.08 | **-0.80** | 0.20 |
| **Dry season rainfall** | 0.36 | 0.46 | 0.49 | -0.20 | -0.02 | 0.18 | 0.32 | 0.00 | 0.33 | 0.40 | -- | 0.19 | -0.20 | 0.22 |
| **Annual Rainfall** | 0.06 | 0.11 | 0.20 | -0.02 | -0.02 | 0.05 | 0.07 | 0.00 | 0.06 | 0.08 | 0.19 | -- | -0.04 | 0.01 |
| **Mean Temp** | -0.29 | -0.61 | -0.52 | 0.36 | -0.23 | -0.24 | -0.48 | -0.22 | -0.56 | -0.80 | -0.20 | -0.04 | -- | -0.14 |
| **Port dist.** | 0.16 | 0.14 | 0.15 | -0.11 | 0.10 | 0.12 | 0.12 | 0.10 | 0.18 | 0.20 | 0.22 | 0.01 | -0.14 | -- |
