# Supplementary material for: Reconciling Oil Palm Expansion and Climate Change Mitigation in Kalimantan, Indonesia
Source: PLoS One. 2015 May 26;10(5):e0127963. doi: 10.1371/journal.pone.0127963 (PMC4444018; doi:10.1371/journal.pone.0127963)
Supplement: S2 Table — (DOCX) [file pone.0127963.s004.docx]

**S2 Table. Variance inflation factors (VIF).** The VIFs indicate the degree to which each variable’s coefficient is inflated by linear dependence with other explanatory variables.

|  | **Variance Inflation Factor** |
| --- | --- |
| **Carbon Stocks** | 1.80 |
| **Distance to concession** | 1.53 |
| **Distance to plantation** | 2.54 |
| **Soil Depth** | 1.42 |
| **Soil Acidity** | 1.25 |
| **Soil Drainage** | 1.68 |
| **Distance to roads** | 1.29 |
| **Distance to rivers** | 1.31 |
| **Slope** | 1.61 |
| **Elevation** | 2.48 |
| **Dry season rainfall** | 4.64 |
| **Annual Rainfall** | 1.15 |
| **Mean Temp** | 1.21 |
| **Distance to ports** | 2.66 |
