# Supplementary material for: Reconciling Oil Palm Expansion and Climate Change Mitigation in Kalimantan, Indonesia
Source: PLoS One. 2015 May 26;10(5):e0127963. doi: 10.1371/journal.pone.0127963 (PMC4444018; doi:10.1371/journal.pone.0127963)
Supplement: S3 Table — (DOCX) [file pone.0127963.s005.docx]

**S3 Table. Parsimonious model specified by iteratively removing the least significant explanatory variable until all variables are significant at P < 0.05.** The model also included 51 district-level dummy variables (not shown). Total number of observations: 5,155. Reported standard errors are Huber-white robust standard errors, clustered by district. P-values are based on a two-sided z-test of the null hypothesis that the parameter estimate equals zero.

|  | **Coefficient** | **Standard Error** | **Pr(>\|z\|)** |
| --- | --- | --- | --- |
| **Intercept** | 1.877 | 0.546 | 0.001 |
| **Distance to concession** | -1.186 | 0.284 | 0.000 |
| **Distance to plantation** | -0.414 | 0.081 | 0.000 |
| **Distance to roads** | -0.679 | 0.302 | 0.025 |
| **Elevation** | -0.088 | 0.017 | 0.000 |
| **Distance to ports** | -0.715 | 0.113 | 0.000 |
