# Supplementary material for: Reconciling Oil Palm Expansion and Climate Change Mitigation in Kalimantan, Indonesia
Source: PLoS One. 2015 May 26;10(5):e0127963. doi: 10.1371/journal.pone.0127963 (PMC4444018; doi:10.1371/journal.pone.0127963)
Supplement: S4 Table — (DOCX) [file pone.0127963.s006.docx]

S4 Table. Comparison of models predicting oil palm plantation expansion based on 2000 – 2005 data and 2005 – 2010 data. Both models include 51 district-level dummy variables as fixed effects. Total number of observations 2000 – 2005: 2,391. Total number of observations 2005 – 2010: 2,761 Reported standard errors are Huber-white robust standard errors, clustered by district. P-values are based on a two-sided z-test of the null hypothesis that the parameter estimate equals zero. Asterisks indicate significance at P < 0.05.

|  | **2000 - 2005 model** | | | **2005 - 2010 model** | | |
| --- | --- | --- | --- | --- | --- | --- |
|  | Coefficient | Standard Error | Pr(>\|z\|) | Coefficient | Standard Error | Pr(>\|z\|) |
| Intercept | -0.534 | 1.873 | 0.885 | -0.858 | 1.897 | 0.651 |
| Distance to existing oil palm concessions (km) | -1.473 | 0.517 | 0.004** | -1.084 | 0.333 | 0.001** |
| Distance to existing oil palm plantations (km) | -0.597 | 0.115 | 0.000* | -0.396 | 0.110 | 0.000*** |
| Distance to major roads (km) | -1.890 | 0.593 | 0.001** | -0.276 | 0.294 | 0.193 |
| Distance to rivers (km) | 0.022 | 0.295 | 0.942 | 0.294 | 0.224 | 0.190 |
| Distance to major ports (km) | -1.012 | 0.269 | 0.000*** | -0.617 | 0.168 | 0.000*** |
| Elevation (km) | -0.039 | 0.070 | 0.577 | -0.091 | 0.027 | 0.001** |
| Slope (%) | -0.028 | 0.028 | 0.308 | -0.028 | 0.027 | 0.309 |
| Annual Rainfall (10 cm) | -0.168 | 0.141 | 0.234 | -0.052 | 0.129 | 0.687 |
| Rainfall in the Driest Quarter (10 cm) | 0.189 | 0.588 | 0.748 | 0.088 | 0.425 | 0.835 |
| Annual Mean Temperature (°C) | 0.054 | 0.129 | 0.676 | 0.015 | 0.080 | 0.851 |
| Soil Depth (cm) | 0.002 | 0.041 | 0.966 | 0.012 | 0.029 | 0.668 |
| Soil Acidity (pH) | -0.042 | 0.024 | 0.089* | 0.097 | 0.123 | 0.434 |
| Soil Drainage Index | 0.370 | 0.173 | 0.032** | 0.146 | 0.138 | 0.289 |
| Biomass Carbon Stocks (10 T C / ha) | -0.012 | 0.027 | 0.186 | -0.037 | 0.020 | 0.058* |
